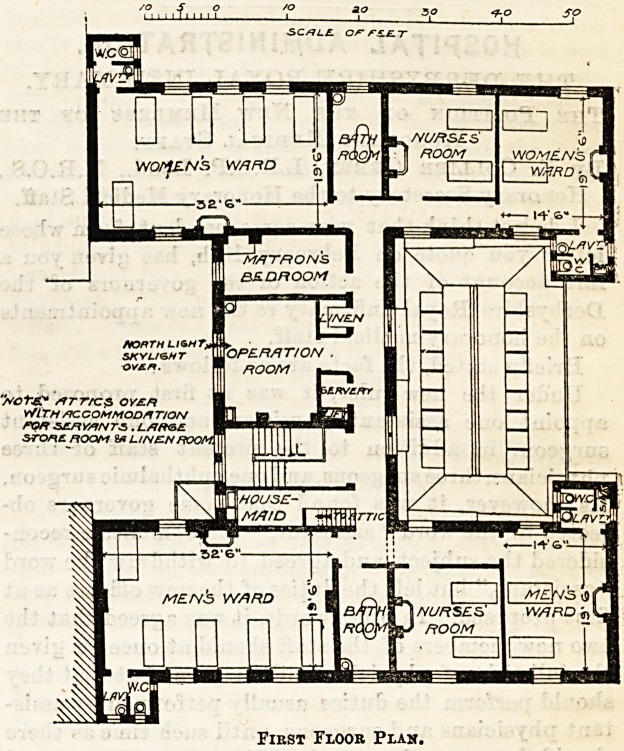# New Eye Infirmary, Sunderland

**Published:** 1895-03-09

**Authors:** 


### THE INSTITUTIONAL WORKSHOP.

### HOSPITAL CONSTRUCTION.

### NEW EYE INFIRMARY, SUNDERLAND.

This building has been erected on a small and apparently confined site in Stockton Street. The out-patient department, always the most important part of an eye hospital, is on the ground floor and has a separate entrance in a side passage. The main entrance to the hospital is in the centre of the street front There is communication at three different points between the out-patient department and the rest of the building, so that the advantage of separating the former entirely has apparently not been thought worth consideration. The out-patients' waiting-hall communicates with the board-room by a large opening which can be closed by a movable wooden screen. The two rooms can be thus thrown together when required for the purpose of holding meetings or concerts. The patients pass from the waiting-hall into the one consulting-room and thence to the dispensary, and leave the building by a separate door. Only one w.c. is provided for out-patients, who presumably are of both sexes; a mistake of a most unaccountable nature. The kitchen offices are on the ground-floor, also a day-room for patients and one for nurses. At the back is a small airing court, off one corner of which is an arrangement we had supposed to be quite obsolete, a large dusthole. The sooner this is converted into an open shed as a shelter for iron dust-bins the better.

Upstairs on the first floor are two men's wards, one

GROUND FLOOR PLAN.

for five patients, the other for two, with a nurses' room for two nurses and a bath-room intervening. At the

FIRST FLOOR PLAN.

back is a precisely similar arrangement, the total number of beds being fourteen. In the centre is the operation-room, which is provided with two somewhat narrow windows and a skylight; the latter, a quite useless arrangement for eye surgery; adjoining on one side is a very small bed-room for the matron, and on the other side the stairs and a housemaid's closet. The size of the matron's bed-room is 8 ft. 6 in. by 17 ft. By narrowing the operation-room and extending it inwards, by abolishing the small linen cupboard and the objectionable lift and servery, the matron's bed-room might have been made of a more suitable size, and the operation-room improved. The plan of placing two nurses in one room is altogether objectionable, and in a building of so recent a date ought to have been avoided. When will hospital committees recognise that a nurse is entitled to some privacy in the course of her life, and that the small amount she can get is entirely denied to her if she has to share her bed-room with another? The attics, not yet furnished, will provide accommodation for staff, and beds for five additional patients.

The drainage and sanitary arrangements have been well planned and carefully carried out, and the wards are furnished with very excellent Teale grates. We see that a so-called automatic system of ventilation has been adopted, involving the use of Tobin tubes and of iron shafts above the ceilings, and depending for its action on so-called extract cowls. We hope, however, that proper provision will be made for window ventilation, as these systems invariably prove to be ineffectual, while there is even less reason for their adoption in an eye hospital, where the patients are for the most part in normal health, than in any other class of hospital.

The building was designed by Mr. J. Wardle Donald, A.R.I.B.A., whose plans were chosen in open competition, and the cost of erection was upwards of £4,700.

## Figures and Tables

**Figure f1:**
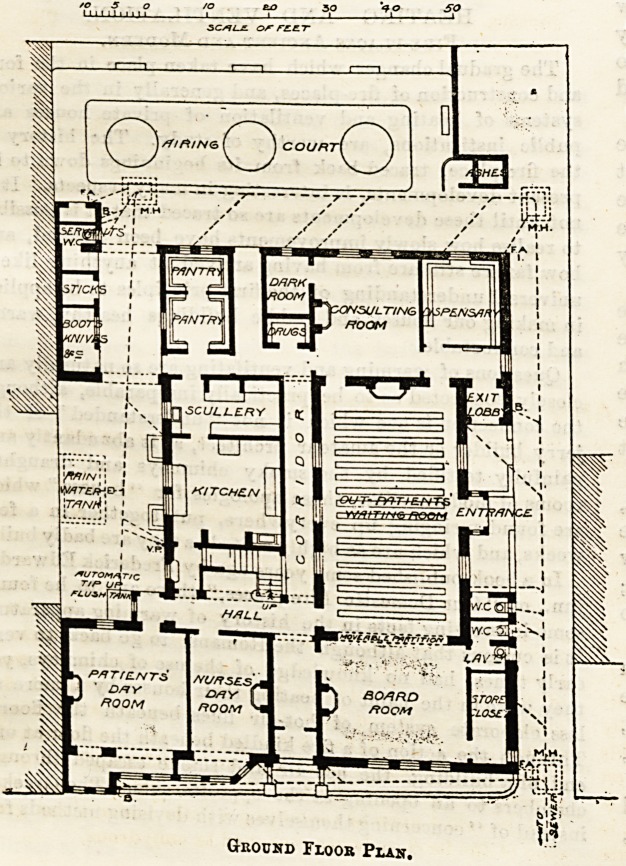


**Figure f2:**